# Gun-Shot Wound—Tetanus—Chloral

**Published:** 1875-01

**Authors:** A. R. Kilpatrick

**Affiliations:** Navasota, Texas


### GUN-SHOT WOUND—TETANUS—CHLORAL.

BY A. R. KILPATRICK, M. D., NAVASOTA, TEXAS.

On the 16th of May, 1874, I was called in the afternoon to see Alexander Harris, three miles in the country. He is aged 14 years, light complexion, good health, and engaged working on a farm.

He was shot by the accidental discharge of his gun, which fell down where he had leaned it against a tree at a distance of five feet from him, and the whole load, with the wadding, passed through his left thigh, tearing away the skin from near the trochanter, downward and inwardly to within three inches of the popliteal region, removing about three pounds in weight of muscle, and exposing the femur for two inches, leaving an ugly, jagged surface all powder-burnt. The hemorrhage was slight and the pulse nearly normal when I saw him, about an hour after the injury. He was very much alarmed, although he bore up bravely under his sufferings. He was carried from the woods where he was shot, on a litter made of poles and a saddle blanket, to his place of residence, over a mile away.

It is needless to detail the minutiæ of the use of chloroform, the application of sutures, etc.

Next day there was considerable fever, requiring refrigerants and cooling dressings. The foot of the wounded limb was so cold as to require covering and artificial warmth for a few days. Used a wash to the wound, of carbolic acid, and gave doses of chlorate potash with sufficient tinct. verat. viridi to control the pulse. On the 19th he was free from fever and continued so several days.

On the 21st added a few grains chloral to the carbolic wash, which was continued during the whole treatment. ℞. Fluid carbolic acid, ʒj.; chloral hydrate, ℈j.; aqua font, ℥iij. Mix. Apply some twice in twenty-four hours.

On the 27th he began to have symptoms of tetanus: stiffness of the jaw, difficulty of chewing food, and pain attending it. This increased so that, on the night of the 29th, the neck was stiff and sore, and during sleep he had quite a spasm, in which he bit his tongue. When I called on the 30th, the rigidity of the neck was quite evident, with tendency to opisthotonos: pains in neck and down the spine and thorax. Can open the jaws but very little, and only partially protrude the tongue. Gave, first, quarter-grain doses of valerianate morphia, repeated *pro re nata.* The tetanic spasms, first more severe at night, but now any sudden noise or jar of the bed produces them. Gave elixir of phos. ferri and calasaya *t. i. d.* and nourishing diet.

On the 31st, had moistened leaves of chewing tobacco applied to the jaws and throat, ard gave twenty grains chloral hydrate every four hours. The beneficial effects of this were soon very apparent. The risus sardonicus was lessened, the pain in the jaws, neck and back relieved, and sleep promoted. Gave always a full dose of chloral a short time before dressing his wound, which enabled him to bear it without suffering. If the chloral is not given regularly and promptly, the tetanic symptoms recur; he will jerk when spoken to, or when he attempts to speak. These jerks begun in the lame leg, and were worse on that side of the body.

In order to avoid causing these sufferings by attempting to masticate, gave him milk, soup, egg-nog, and all his diet in fluid form, and he sucked it through a large-sized glass tube. Gave the chloral dissolved in camphor water.

On the 8th of June the tetanic symptoms are much milder; can open the jaws wider, protrude the tongue; but cannot chew any. Continue the chloral, both to sore and by draft, and also the tobacco leaves to the jaws. When he has the spasm the wound gapes open and deranges the dressings. This is in consequence of the divided muscles pulling in opposite directions.

On the 13th was worse; more convulsive. Increased the chloral and gave twenty grains bromide potash with it. Administered chloroform by inhalation in order to dress his wound. This quieted him and relaxed the jaws very well. Add quinine to elixir calasaya.

On the 18th his improvement was perceptible. Tetanic symptoms milder and sleeps better; good appetite.

On the 20th have iodoform dusted thrice over the surface of the sore, after washing it.

On the 26th his improvement was such as to require the chloral and bromide only at night. All his symptoms denoted improvement; the sore was filling up, granulating and closing up well. Dress the sore with iodoform in glycerin and olive oil, once a day.

July 1st, discontinue the use of chloral and bromide. On the 2d he is able to get up and stand on his feet, but the left foot is very numb, and soon becomes so swollen and painful as to force him to lie down. Tetanus continued thirty-two days.

From this time I saw him only occasionally. His sore continued to heal and he was up and about the house and yard. By the last of August the sore was entirely healed. He is now at work; walks without limping, and rides horseback as if he had never been hurt.

This case may be added to others, showing the beneficial agency of chloral hydrate in the treatment of tetanus.

